# Antifungal activity of the nucleoside mimetic 5-fluorouridine against *Candidozyma auris* and *Candida* species

**DOI:** 10.1128/aac.00027-26

**Published:** 2026-08-14

**Authors:** Bharath Reddy Boya, Ezhaveni Sathiyamoorthi, Jin-Hyung Lee, Jintae Lee

**Affiliations:** 1 School of Chemical Engineering, Yeungnam University 35032, Gyeongsan, Republic of Korea; University Children's Hospital Münster, Münster, Germany

**Keywords:** antifungal, *Candida*, cytidine, fungi, nucleoside, uridine

## Abstract

The emergence of multidrug-resistant *Candidozyma auris* has created an urgent need for antifungals with novel mechanisms of action. We screened 121 nucleoside and nucleobase derivatives against *C. auris* and other clinically relevant *Candida* species to identify effective antimetabolite scaffolds. Among all compounds tested, only the C-5-fluorinated nucleosides 5-fluorouridine (5-FU) and 5-fluorocytidine exhibited potent antifungal activity. 5-FU demonstrated consistently low minimum inhibitory concentrations (0.5–10 µg/mL) across multiple *Candida* species, including fluconazole-resistant strains, and displayed fungicidal activity against *C. auris* at concentrations lower than several frontline antifungals. Uridine rescue assays confirmed competitive interference with endogenous uridine metabolism. Checkerboard assays revealed synergistic or additive interactions between 5-FU and clinically used azoles and echinocandins. Toxicity evaluation using plant, nematode, and human cell models showed minimal cytotoxicity even at concentrations far exceeding antifungal minimum inhibitory concentrations. *In silico* docking analyses revealed dual inhibition of thymidylate synthase and RNA polymerase III, explaining the fungicidal phenotype and broad-spectrum efficacy. These findings identify 5-fluorouridine as a promising antifungal candidate and establish C-5-halogenated nucleosides as a privileged scaffold for antifungal drug development.

## INTRODUCTION

Invasive *Candida* infections pose a severe threat to global public health. Candidiasis is a condition where the *Candida* propagates and grows uncontrollably in the infection site (1). Candidiasis can affect various parts of the body, including skin, mouth (oral candidiasis or thrush), vagina (vaginal candidiasis), and bloodstream (invasive candidiasis) (2). Candidiasis can be caused by various species of the *Candida* genus, such as *Candida glabrata*, *Candida parapsilosis*, and *Candidozyma auris*, but *Candida albicans* remains the primary contributor, with 46% of the reported cases. Widespread use of antifungal prophylaxis and the resulting selective pressure on fungal populations leads to increased infections of non-albicans species (3). Global estimates suggest that 250,000–700,000 cases of candidiasis are reported annually, and a majority of the annual 1.7 million fungal infection deaths are attributed to the *Candida* species (4). The challenge of antifungal resistance has been intensified by the emergence of *Candidozyma auris* (formerly *Candida auris*), a multidrug-resistant yeast first identified in 2009 (5). It rapidly spreads in healthcare settings due to its persistence on surfaces, high transmissibility, and resistance to major antifungal classes, including azoles, amphotericin B, and echinocandins (5). *C. auris* adheres to skin and medical devices via adhesins, evades host immunity through β-glucan masking and reduced neutrophil response, and exhibits stress tolerance that supports environmental survival (6). Its biofilm formation and multidrug resistance highlight the urgent need for novel antifungal strategies (7).

Nucleosides and their analogs have long served as versatile therapeutic scaffolds, with several showing potent antimicrobial and antiviral activity (8), for example, acyclovir targets herpesviruses, ribavirin exhibits broad-spectrum antiviral effects, and cordycepin (3′-deoxyadenosine) demonstrates strong antifungal activity by disrupting polyadenylation in *Candida* and *Aspergillus* species (9). Nucleoside analogs mimic the function of endogenous nucleosides, thereby disrupting cellular metabolism by incorporating into nucleic acids (8). Anticancer and antiviral analogs usually include modifications like halogenation, methylation, and hydroxylation or dihydroxylation (8). Fluorinated pyrimidines were first synthesized for antitumor activity but were later repurposed for their antifungal activity (10). From this family, 5-fluorouracil has shown promise as an antimicrobial agent but is also toxic to eukaryotic cells (11). Another fluorinated pyrimidine, 5-fluorocytosine, also showed potent antifungal activity against *C. albicans* and *Cryptococcus* by deaminating to 5-fluorouracil first and then metabolizing to floxuridine triphosphate, thereby preventing thymidylate synthase catalysis (8). As eukaryotic cells lack the deaminase for 5-fluorouracil conversion, 5-fluorocytosine was deemed safe for antifungal medication (12). The fluorine atom is a key structural modification that acts as an antimetabolite, disrupting fungal nucleic acid synthesis pathways (13). Since halogenation improves antimicrobial activity and selective halogenation improves the toxicity profile of the scaffolds (14, 15), both phenomena observed in the case of the 5-fluorocytosine, halogenated nucleoside, and nucleobase analogs have the potential to combat *C. auris* infections.

Naturally occurring nucleosides such as uridine, cytidine, adenosine, and guanosine form the biochemical backbone of RNA and are also well-known for their antimicrobial derivatives (8). The modification at the C-5 position of the pyrimidine ring of nucleosides is the most pivotal and potent for antimicrobial activity (10). Based on this insight, we tested C-5 halogenated nucleoside analogs against *C. auris* for their antifungal activity. 5-Fluorouridine (5-FU) emerged as the most potent C-5-halogenated nucleoside, with 5-fluorocytidine (5-FC) also exhibiting strong antimicrobial activity. 5-Fluorouridine is a metabolite of 5-fluorouracil and is used in laboratory settings for RNA labeling (16). 5-Fluorouridine monophosphate, as a result of metabolization of 5-fluorouracil, was previously reported to be active against *C. albicans* (17). Therefore, we studied 5-fluorouridine as a potential antifungal agent against *C. auris* and other *Candida* species (17). In this study, we investigate the antifungal efficacy of 5-fluorouridine against *C. auris* and other *Candida* species by assessing its impact on cell growth and survival. We further examine its potential as an adjuvant by evaluating synergistic interactions with clinically used antifungals, supporting a combinatorial therapeutic approach. To elucidate its mechanism of action, we conduct *in silico* molecular docking and interaction analyses targeting thymidylate synthase and RNA polymerase enzymes of various *Candida* species. Additionally, we assess the toxicological profile of 5-fluorouridine through nematode, plant, and human cell line assays, complemented by *in silico* adsorption, distribution, metabolism and elimination (ADME) predictions, to determine its suitability for therapeutic development.

## MATERIALS AND METHODS

### Nucleoside derivatives

Nucleosides and their derivatives were purchased from Combi Blocks (San Diego, CA, USA). All the chemicals used in the study had a purity percentage ≥98%. The chemicals were dissolved in dimethyl sulfoxide (DMSO) to prepare 100 mg/mL stocks and were refrigerated at −20°C. The final DMSO concentration was equal to or below the recommended 0.1% per mL in the experimental samples, which had a negligible effect on fungal growth or virulence. All the nucleoside and nucleobase derivatives studied are listed in Table S1 with their respective CAS numbers.

### Fungal growth conditions

Fungal strains belonging to the *Candida* genus were used in this study and were obtained either from the Korean Culture Center for Microorganisms (KCCM) or the American Type Culture Collection (ATCC; Manassas, VA, USA). The selected *Candida* panel encompasses both conventional and emerging pathogens with distinct virulence strategies and antifungal resistance mechanisms, thereby providing a clinically relevant framework to assess the broad-spectrum efficacy of the tested compounds. For *C. albicans*, the multi-azole-resistant strain DAY185 and the fluconazole-susceptible strain ATCC 10231 were used. For *C. auris*, strains KCTC 17809 and KCTC 17810 isolated from middle ear infections were used. For *C. glabrata*, ATCC2001 and KCCM50701 strains were used, whereas for *C. parapsilosis*, ATCC7330, ATCC22019, and KCCM50030 were used. Glycerol stocks of all the strains were streaked onto potato dextrose agar (PDA) plates and incubated at 37°C for 48 h to form single colonies (18). As previously described (19), for *C. albicans* and *C. auris*, a single colony was inoculated into 25 mL of potato dextrose broth (PDB) and tryptic soy broth (TSB) in Erlenmeyer flasks at 37°C at 250 rpm for 48 h. For *C. glabrata* and *C. parapsilosis*, a single colony was inoculated into 2 mL of liquid yeast malt medium supplemented with 2% glucose in 14 mL Falcon tubes at 37°C and 250 rpm for 48 h. Overnight cultures hereafter are referred to as the cultures grown at 37°C and 250 rpm for 48 h.

### Minimum inhibitory concentrations and cell growth assay of nucleoside derivatives and antifungal compounds

Planktonic cell growth was assessed using a microplate reader (Multiskan SkyHigh microplate reader; Thermo Fisher Scientific, Waltham, MA, USA) (19). Minimum inhibitory concentrations (MICs) were determined based on the Clinical and Laboratory Standards Institute guidelines using the broth dilution method. Overnight cultures of *C. auris* and *C. albicans* were diluted in TSB and PDB, respectively, at 10^6^ colony-forming unit (CFU)/mL with or without the nucleoside derivatives and antifungal compounds at concentrations of 0.5, 1, 2, 5, 10, 20, 50, 100, 250, and 500 µg/mL in 96-well plates (SPL Life Sciences, South Korea) and incubated at 37°C for 24 h. MIC was considered as the lowest concentration at which no visible growth was observed. For cell growth curves, *C. auris* KCTC 17809 overnight cultures were diluted to 10^5^ CFU/mL with or without 5-FU and 5-FC at 0.025, 0.5, 1, and 5 µg/mL in 96-well plates and incubated at 37°C for 24 h. Absorbances were recorded at 600 nm at 0, 2, 4, 6, 8, 10, and the 24 h of the incubation.

### Cell survival assay and uridine rescue assay

Cell survival assay was performed as previously described (18, 20) by diluting overnight cultures of the *C. auris* KCTC 17809 to 10^5^ CFU/mL with or without 5-FU and 5-FC at 0.5, 2.5, 10, and 20 µg/mL and antifungal compounds at their MIC and 5× MIC concentrations. The cultures were incubated at 37°C with shaking at 250 rpm. At 0, 1, 2, 3, 4, and the 24 h, cultures were serially diluted, plated on PDA plates, and incubated at 37°C for 24 h. The CFUs were counted post 24 h incubation of the agar plates.

Uridine rescue assay was performed as previously described with minor modifications (21). *C. auris* KCTC 17809 overnight cultures were diluted to 10^5^ CFU/mL with or without 5-FU and 5-FC at 0.025, 0.5, 1, and 10 µg/mL, and an equal amount of uridine was added in 96-well plates and incubated at 37°C for 24 h. Absorbances were recorded at 600 nm post 24 h incubation.

### Checkerboard assay

To assess the synergistic potential of 5-FU and 5-FC with mainstream antifungals, a checkerboard assay was done (22). Overnight cultures of *C. auris* KCTC 17809 were diluted to 10^5^ CFU/mL in TSB with or without the combination of nucleosides 5-FU and 5-FC and the antifungals clotrimazole, econazole, and miconazole, and incubated at 37°C for 24 h. Planktonic growth was measured by reading absorbance at OD600. The concentration completely inhibiting growth by absorbance and visual inspection was deemed an inhibitory combination. The concentration series tested for 5-FC and 5-FU were 0, 0.1, 0.2, 0.3, 0.4, 0.5, and 1 µg/mL. For antifungals, the final concentrations were clotrimazole (0, 2, 4, 6, 8, 10, and 12 µg/mL), econazole (0, 2, 3, 4, 5, 6, and 8 µg/mL), and miconazole (0, 0.5, 1, 1.5, 2, 3, and 5 µg/mL). Fractional inhibitory concentrations (FICs) were determined, and fractional inhibitory concentration indices (FICIs) were calculated to assess drug interactions using the formula:


FICI=(FIC1/MIC1)+(FIC2/MIC2)

Interaction outcomes were interpreted based on FICI values, where ≤0.5 indicates synergy, 0.5–1 indicates an additive effect, 1–2 indicates an indifferent interaction, and values > 2 indicate antagonism.

### Reactive oxygen species production assay

Reactive oxygen species (ROS) assay was performed as previously described (23). Cells from overnight cultures of *C. auris* KCTC 17809 were harvested and resuspended to 10^6^ CFU/mL in phosphate-buffered saline and treated with or without 5-FU, 5-FC (0.5 and 2 µg/mL), and H_2_O_2_ (1,000 and 2,000 µg/mL) as a positive control for 1 h at 37°C and 250 rpm. The OD600 absorbances of cell suspensions were measured and treated with 2′,7′-dichlorofluorescein diacetate (20 μM) and incubated in the dark for 30 min at 37°C. Fluorescence measurements were taken using an F-7000 spectrofluorometer (Hitachi, Tokyo, Japan) with a xenon arc lamp. The excitation and emission slits were set to 5 nm, with an excitation wavelength of 510 nm, and emission intensities recorded at 524 nm. Fluorescence intensities were normalized by OD600 values to account for cell growth.

### Toxicity assessment using *C. elegans* nematode model

*C. elegans* strain *fer-15(b26); fem-1(hc17*) was used as the cytotoxicity model as previously described (22). Synchronized adult worms (*n* = 30 per well) were suspended in M9 buffer with or without 5-FU and 5-FC at concentrations of 5, 20, 100, and 200 µg/mL in 96-well plates and incubated at 25°C for 7 days. Worm survival was assessed by their motility in response to blue LED light exposure for 20 s using an iRiS Digital Cell Imaging System (Logos Biosystems, Anyang, South Korea).

### Phytotoxicity assessment using a seedling growth model

Phytotoxicity was assessed by measuring seedling length of *Brassica rapa* (winter cabbage) seeds as previously described (22). Seeds were assessed and sorted for defects, washed, and soaked in distilled water for 8 h, and air-dried overnight. Ten seeds were placed on Murashige and Skoog (0.86 g/L) soft agar (0.7%) plates with or without 5-FU and 5-FC at 1, 5, 20, and 100 µg/mL. The plates were incubated at 25°C for 5 days. The total length of the germinated seedlings was measured daily.

### Cytotoxicity assessment using MTT assay of HepG2 cell line

Cytotoxicity assessment using HepG2 cell line was performed as previously described (24). Human hepatocellular carcinoma HepG2 cell lines were procured from the Korean Cell Line Bank. The cell lines were cultured in DMEM medium supplemented with 10% fetal bovine serum (Gendepot, Texas, USA) in a 96-well plate in a 5% CO_2_ incubator at 37°C for 24 h. Post incubation, the spent medium was removed and fresh medium with or without 5-FU and 5-FC at 20, 100, 300, 1,000, and 3,000 µg/mL was added and incubated further for 24 h. For the MTT assay, the culture medium was aspirated using a vacuum, and 100 µL of fresh medium containing MTT (final concentration: 0.5 mg/mL; prepared by a 1:10 dilution of stock solution) was added to each well. The plates were then incubated at 37°C in a humidified 5% CO_2_ incubator for 4 h to allow for formazan crystal formation. Following incubation, the supernatant was carefully removed, and 100 µL of DMSO was added to each well to dissolve the formazan crystals under dark conditions. Absorbance was then measured at 540 nm to determine cell viability.

### *In silico* analysis: molecular docking, multiple sequence alignment, and in silico ADMET

Molecular docking analysis of thymidylate synthase of *Candida* spp. was performed using the glide module of Maestro 12.5 (Schrodinger Software Solutions, United States). AlphaFold (DeepMind, United Kingdom) was used to predict protein structure of thymidylate synthase of *C. auris* (UniProt entry A0A0L0P707), *C. parapsilosis* (UniProt entry G8BCL7), *C. glabrata* (UniProt entry Q0Z9A6), and *C. albicans* (UniProt entry P12461). Protein structure validation was done using PROCHECK (https://github.com/RomanLas/PROCHECK) and ERRAT tool (https://github.com/liueic/ERRAT-Rust) in the UCLA Saves server. Colabfold was used to predict protein structure of RNA polymerase (subunits RPC1 and RPC2 to form elongation complex) of *C. albicans* (GenBank entry RPC1-KAM3121573.1 and RPC2-KHC77882.1) and *C. auris* (GenBank entry RPC1-KHC86330.1 and RPC2-KAM3119593.1). Structures with confidence scores above 90% threshold were chosen.

The 3D structures of the ligand compounds were drawn in ChemDraw Ultra 12.0 (Revvity Signals Software, Massachusetts, USA), and their SMILES strings were extracted. The SMILES strings of the compounds were converted into 3D SDF files using the NovaPro web application (https://www.novoprolabs.com/tools/smiles2pdb, accessed on 6 August 2025). The protein was prepared using the protein preparation wizard, and ligands were prepared using LigPrep and minimized using OPLS3e force field. From literature search and UniProt, the essential binding site residue for thymidylate synthase was determined as Cys176 (Cys175 for *C. glabrata*) in the catalytic pocket, which was chosen as the centroid for grid generation. For RNA polymerase docking, an essential binding site motif was identified as NADFDGD motif (505–511) in the catalytic pocket, which was chosen as the centroid for grid generation. Two MG^2+^ metal coordination sites at stable bond order were inserted in the grid to replicate RNA polymerase elongation. Both grids also coincided with the best binding site identified by the sitemap analysis in Maestro 12.5 (results not shown). Glide XP docking (extra precision) was performed for the protein and ligands with three-pose limit, and the best pose was chosen for analysis and docking score evaluation.

ADMET profiles (adsorption, distribution, metabolism, elimination, and toxicity) of 5-FC and 5-FU were evaluated using online webservers PreADMET (https://preadmet.webservice.bmdrc.org/) and ProTox 3.0 (https://tox.charite.de/protox3/) (accessed on 12 December 2025).

### Statistical analysis

All experiments were performed using at least two independent cultures in triplicate samples. Data are presented as mean ± standard deviation. Statistical significance was determined using Student’s *t*-test, and *P*-values ≤ 0.05 were considered statistically significant. All graphs were generated using SigmaPlot version 14.0.

## RESULTS

### Antifungal activities of nucleoside derivatives against *C. auris*

Initial assessment of the antifungal activities of the nucleoside derivatives was done by screening 121 nucleosides and nucleobases (Table S1) against *C. auris* KCTC 17809 and *C. albicans* DAY185. All the derivatives except 5-FU and 5-FC did not show any inhibition of note (>500 µg/mL) in both *C. auris* and *C. albicans* (results not shown). Both 5-FU and 5-FC showed MICs of 0.5 µg/mL against *C. auris* (Table 1). Against fluconazole-resistant *C. albicans* DAY185, 5-FU and 5-FC showed MICs of 1 and 20 µg/mL, respectively (Table 1). Based on their activity against *C. auris* and *C. albicans*, 5-FU and 5-FC were chosen as hits for further investigation. 5-FU was active against all nine *Candida* strains tested, with MICs lower than 20 µg/mL. While 5-FC had varied activity against different *Candida* strains, with MICs ranging from 0.5 to >500 µg/mL. They were mostly ineffective against *C. glabrata* and *C. parapsilosis* while being potent against *C. auris* as well as *C. albicans* (Table 1).

**TABLE 1:** MICs of 5-fluorocytidine and 5-fluorouridine against various *Candida* species

Strains	5-Fluorocytidine (µg/mL)	5-Fluorouridine (µg/mL)
*C. albicans* DAY185	20	1
*C. albicans* ATCC10231	200	8
*C. glabrata* ATCC2001	>500	20
*C. glabrata* KCCM50701	>500	20
*C. parapsilosis* ATCC7330	>500	10
*C. parapsilosis* KCCM50030	400	9
*C. parapsilosis* ATCC22019	>500	10
*C. auris* KCTC17809	0.5	0.5
*C. auris* KCTC17810	0.5	0.5

**Fig 1:**
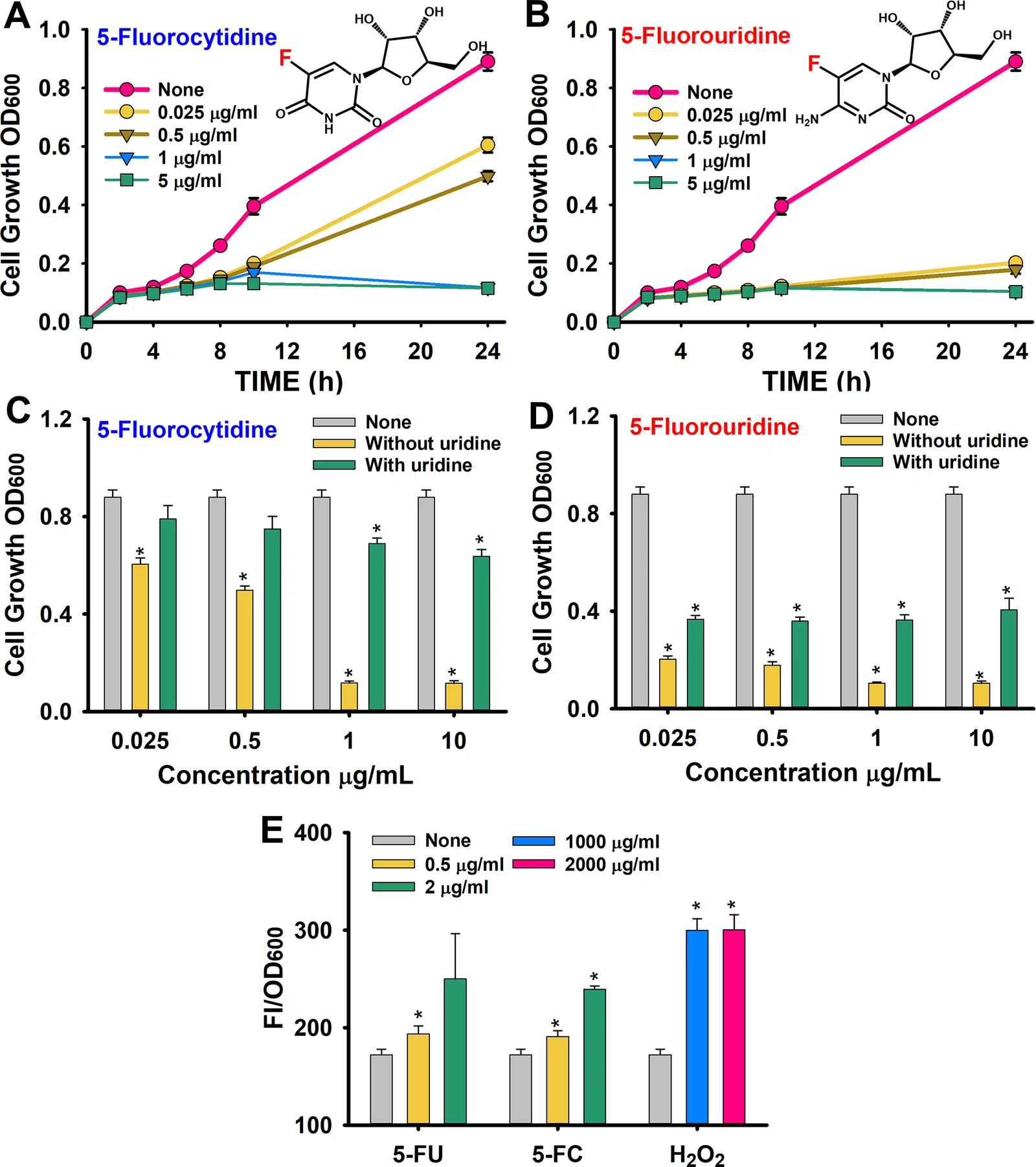
Cell growth curves of *C. auris* against 5-fluorocytidine (**A**) and 5-fluorouridine (**B**). Uridine rescue assay 5-fluorocytidine (5-FC) (**C**) and 5-fluorouridine (5-FU) (**D**). ROS production assay was performed to assess oxidative stress induced by 5-FU, 5-FC, and H_2_O_2_ as a positive control (**E**). *P < 0.05 vs. non-treated controls.

The cell growth curves of *C. auris* against 5-FC and 5-FU show dose-dependent growth inhibition. While sub-MIC (0.025 µg/mL) shows significant growth inhibition, MIC and supra-MICs (>0.5 µg/mL) were able to completely inhibit cell growth (Fig. 1A and B). 5-FU was more effective at cell growth inhibition than 5-FC at 0.025 µg/mL (Fig. 1A and B). As uridine has the ability to mitigate cytotoxicity induced by 5-halogenated nucleosides and nucleobases like 5-fluorouracil (25), we performed a uridine rescue assay against *C. auris* to see if native substrate uridine is able to reverse the antifungal activities of 5-FC and 5-FU. Uridine dose-dependently rescued *C. auris* cells when administered in an equal concentration to that of 5-FC and 5-FU (Fig. 1C and D), signified by an increase in absorbance at OD_600_. At 20× MIC treatment of 5-FC and 5-FU, uridine (similar concentration) was able to restore ~90% and ~50% cell growth, respectively.

**Fig 2:**
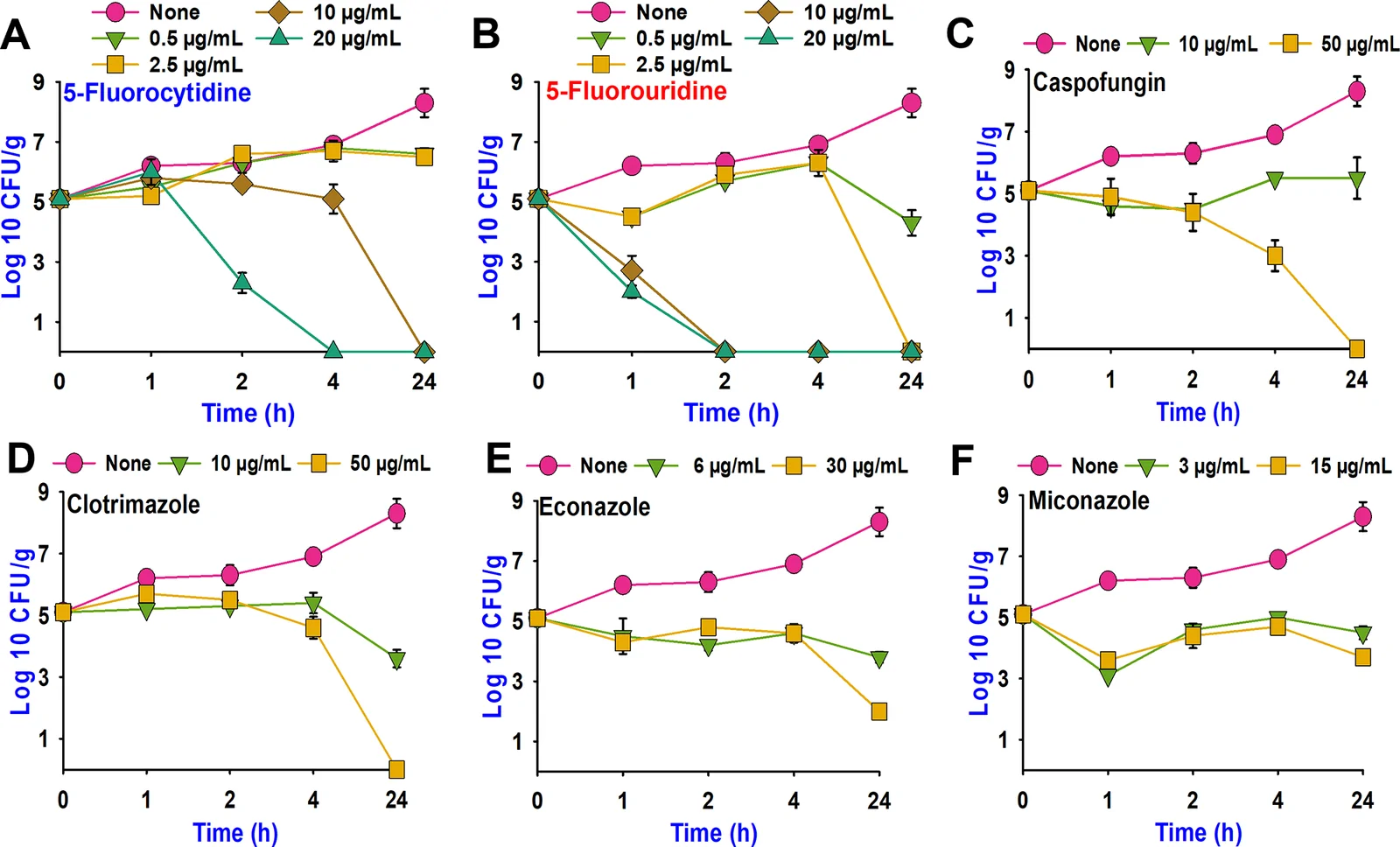
Cell survival assay of *C. auris* against 5-fluorocytidine (**A**), 5-fluorouridine (**B**), caspofungin (**C**), clotrimazole (**D**), econazole (**E**), and miconazole (**F**).

The ROS production assay was performed to check the ability of the nucleoside derivatives to increase intracellular ROS levels, leading to cell mortality. 5-FU and 5-FC did not increase intracellular ROS levels significantly at MIC. But at 4× MIC, there was a significant increase in ROS production, at par with the positive control H_2_O_2_ at 1,000 µg/mL (Fig. 1E). Cell survival assay was performed to assess the killing efficacy of the fluorinated nucleosides. Both 5-FC and 5-FU were fungicidal against *C. auris*. The minimum fungicidal concentration (MFC) of 5-FC and 5-FU was 10 and 2.5 µg/mL (Fig. 2A and B). This is significantly lower in comparison to the clinical antifungals tested, like caspofungin and clotrimazole (both had MFC of 50 µg/mL; Fig. 2C and D). Econazole and miconazole were both fungistatic at the 5× MIC against *C. auris* (Fig. 2E and F). The clinical antifungals demonstrated their published characteristic of fungicidal and fungistatic behavior.

### Synergy of nucleoside derivatives with clinical antifungals against *C. auris*

**Fig 3:**
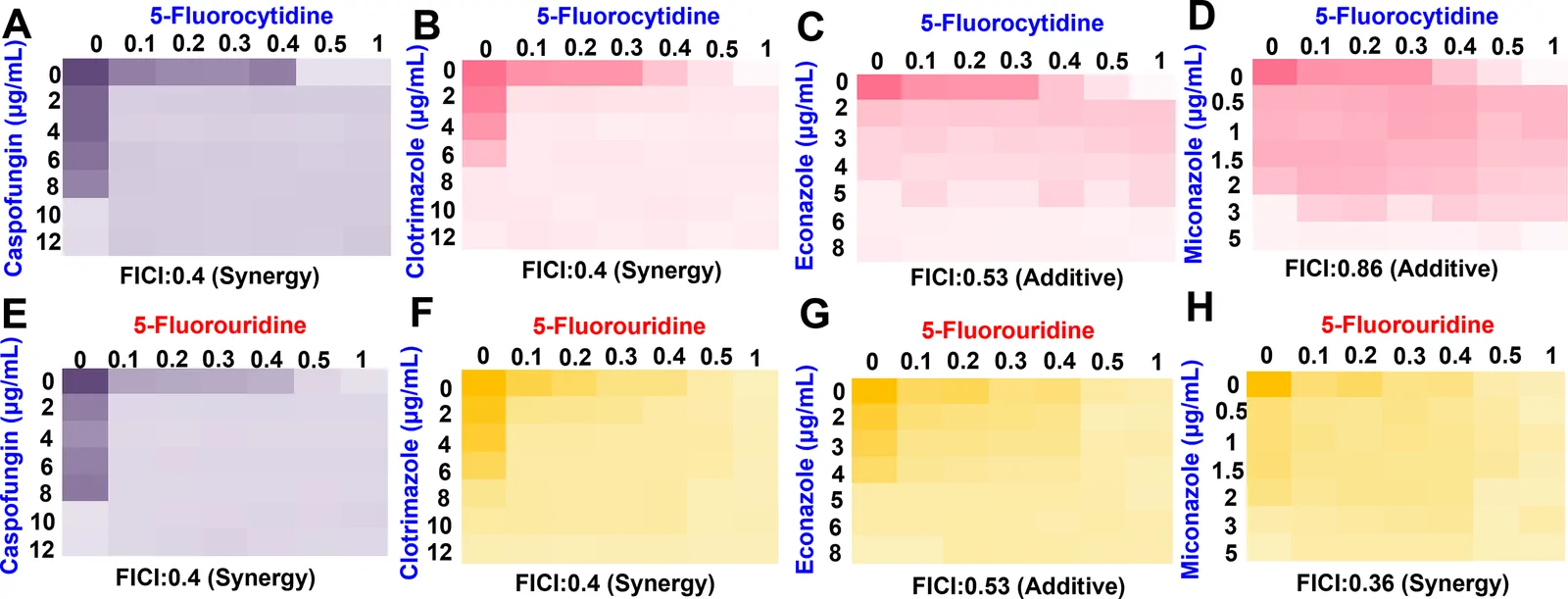
Checkerboard assay of 5-fluorocytidine in combination with caspofungin (**A**), clotrimazole (**B**), econazole (**C**), and miconazole (**D**). Checkerboard assay of 5-fluorouridine in combination with caspofungin (**E**), clotrimazole (**F**), econazole (**G**), and miconazole (**H**).

A checkerboard assay was performed to evaluate the combinatory therapeutic efficacy of popular clinical antifungals and the nucleoside derivatives against the planktonic cell growth of *C. auris*. The MICs of compounds belonging to popular clinical antifungal classes like polyenes, azoles, and echinocandins were evaluated against *C. auris* (Table S2 ). Of the tested antifungals, caspofungin, clotrimazole, econazole, and miconazole were chosen for the checkerboard assay, based on the diverse modes of action and relatively lower MIC values. 5-FC showed synergistic activity with caspofungin and clotrimazole, with FICI values >0.4, whereas they exhibited additive activity with econazole and miconazole, with FICI values ranging from 0.5 to 0.9 (Fig. 3A through 3D). 5-FU also exhibited synergy with caspofungin, clotrimazole, and miconazole but had only additive effects with econazole (Fig. 3E through 3H).

### Toxicity of the nucleoside derivatives

**Fig 4:**
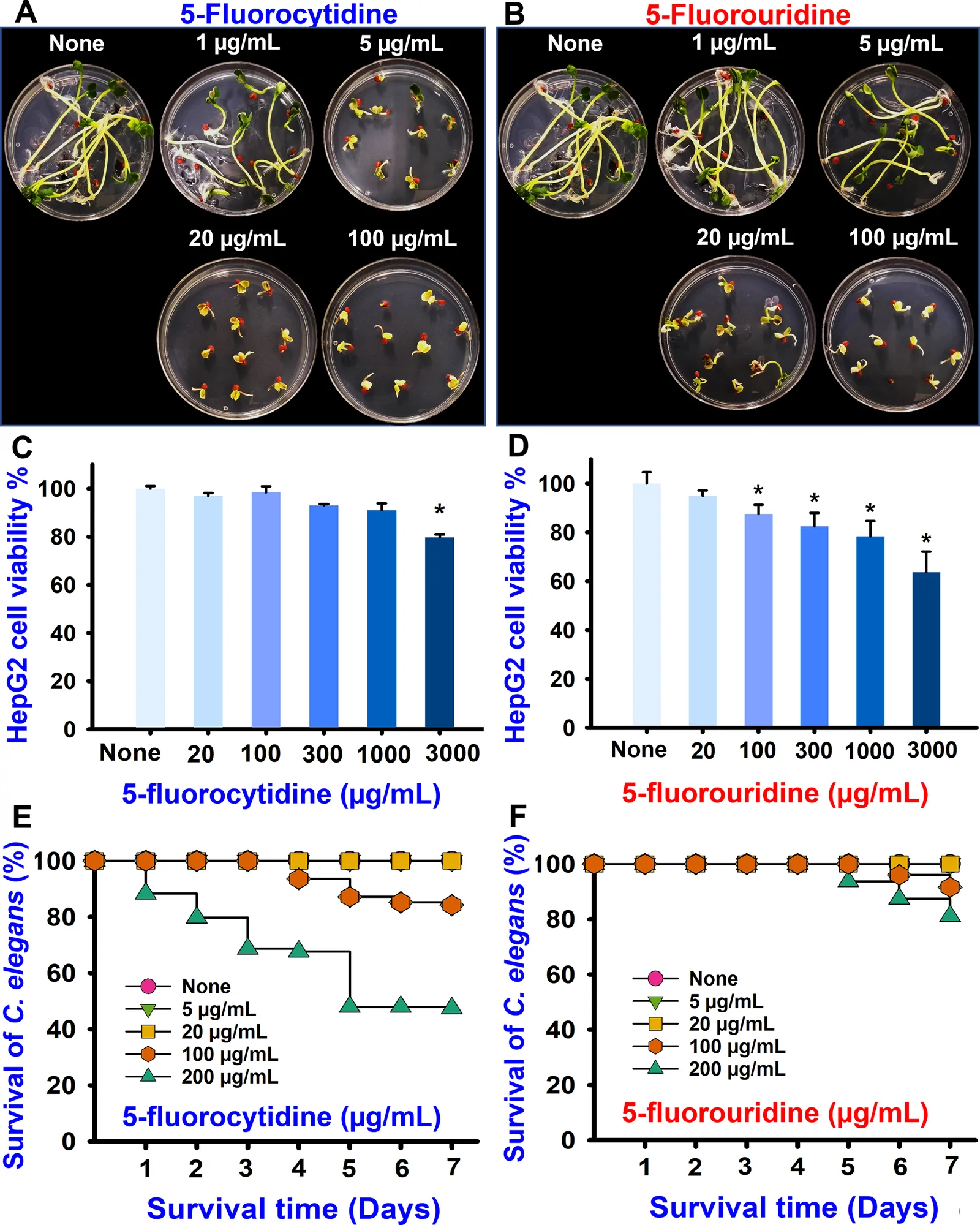
Toxicity of the nucleosides was assessed using *Brassica rapa* seed germination model using the Murashige and Skoog agar medium at 25°C for 7 days. The seed germination of seeds on treatment with 5-fluorocytidine (**A**) and 5-fluorouridine (**B**). Cytotoxicity of 5-fluorocytidine (**C**) and 5-fluorouridine (**D**) against HepG2 cells. *C. elegans* cytotoxicity of 5-fluorocytidine (**E**) and 5-fluorouridine (**F**). *P < 0.05 vs. non-treated controls.

To assess the toxicity and clinical suitability of the nucleoside derivatives, they were tested in phytotoxicity, nematode, and human cell line models in addition to an *in silico* ADMET analysis. Both 5-FC and 5-FU did not show any phytotoxicity to the *Brassica rapa* seeds at 2× MIC concentration of 1 µg/mL (Fig. 4A and B). They did not affect the seed germination as well as the seedling lengths in comparison to the non-treated sample. At 5 µg/mL, 5-FU did not show any effect on seed germination but limited the seedling length by a small margin, whereas 5-FC had no effect on seed germination but completely inhibited seedling growth (Fig. 4A and B). This effect was also observed for both 5-FC and 5-FU at higher concentrations of 20 and 100 µg/mL (Fig. 4A and B).

The viability of the human HepG2 cell line on treatment of the nucleoside derivatives was tested. Remarkably, both 5-FC and 5-FU showed survival percentages of >60% at an extreme concentration of 3,000 µg/mL (6,000× MIC; Fig. 4C and D). The lowest concentration tested for both compounds was 20 µg/mL, and even at this concentration (40× MIC), the survival percentage was above 90%, signifying extremely low toxicity of the nucleoside compounds against the human HepG2 cell line (Fig. 4C and D). Furthermore, the two compounds were subjected to toxicity testing using the *C. elegans* nematode model, an established model for compound toxicity testing (26, 27). Both 5-FC and 5-FU did not affect worm survival at 20 µg/mL (Fig. 4E and F). At 100 µg/mL, 5-FU-treated worms had worm survival of ~90%, whereas 5-FC-treated worms had worm survival of ~80% (Fig. 4E and F). At 200 µg/mL (400× MIC), 5-FU exhibited worm survival >80%, whereas 5-FC showed a more moderate survival of 50% (Fig. 4E and F). Both the nucleoside derivatives were non-toxic to *C. elegans* worms. *In silico* ADMET analysis showed positive toxicity and ADME profile. While the predictions showed some systemic toxicity of the compounds (hepatotoxicity, nephrotoxicity, and carcinogenicity), which warrants further investigation, they did not exhibit any further toxicity to other systems (Table S3). Remarkably, both 5-FU and 5-FC were inactive against all the Tox-21 parameters tested (Table S3). They also demonstrated good ADME properties like organ permeability, logP values, and non-Pgp inhibition. They did not violate any Lipinski’s rule of five and were qualified under the World Drug Index rule (Table S3).

### Molecular docking of nucleoside derivatives against thymidylate synthase and RNA polymerase III

The nucleoside derivatives 5-FU and 5-FC are structurally similar to 5-fluorouracil and 5-fluorocytosine. Based on similar inhibitory activities, we envisaged that both nucleoside derivatives might have a similar mode of action of nucleic acid synthesis inhibition, as that of 5-fluorouracil and 5-fluorocytosine against *Candida* species. This would be the inhibition of the thymidylate synthase (DNA synthesis inhibition) and RNA polymerase III elongation (RNA synthesis) (28, 29). Therefore, a molecular docking analysis was performed with the thymidylate synthase and RNA polymerase III RPC1 and RPC2 elongation complex of the *Candida* species and the metabolized products of the nucleoside derivatives. 5-FC and 5-FU can be metabolized to an active 5-fluoro-uridine triphosphate (5-FUTP) and 5-fluorodeoxyuridine monophosphate, acting in the RNA and DNA synthesis pathways, respectively. A detailed description of the mode of action is provided in the subsequent section.

**Fig 5:**
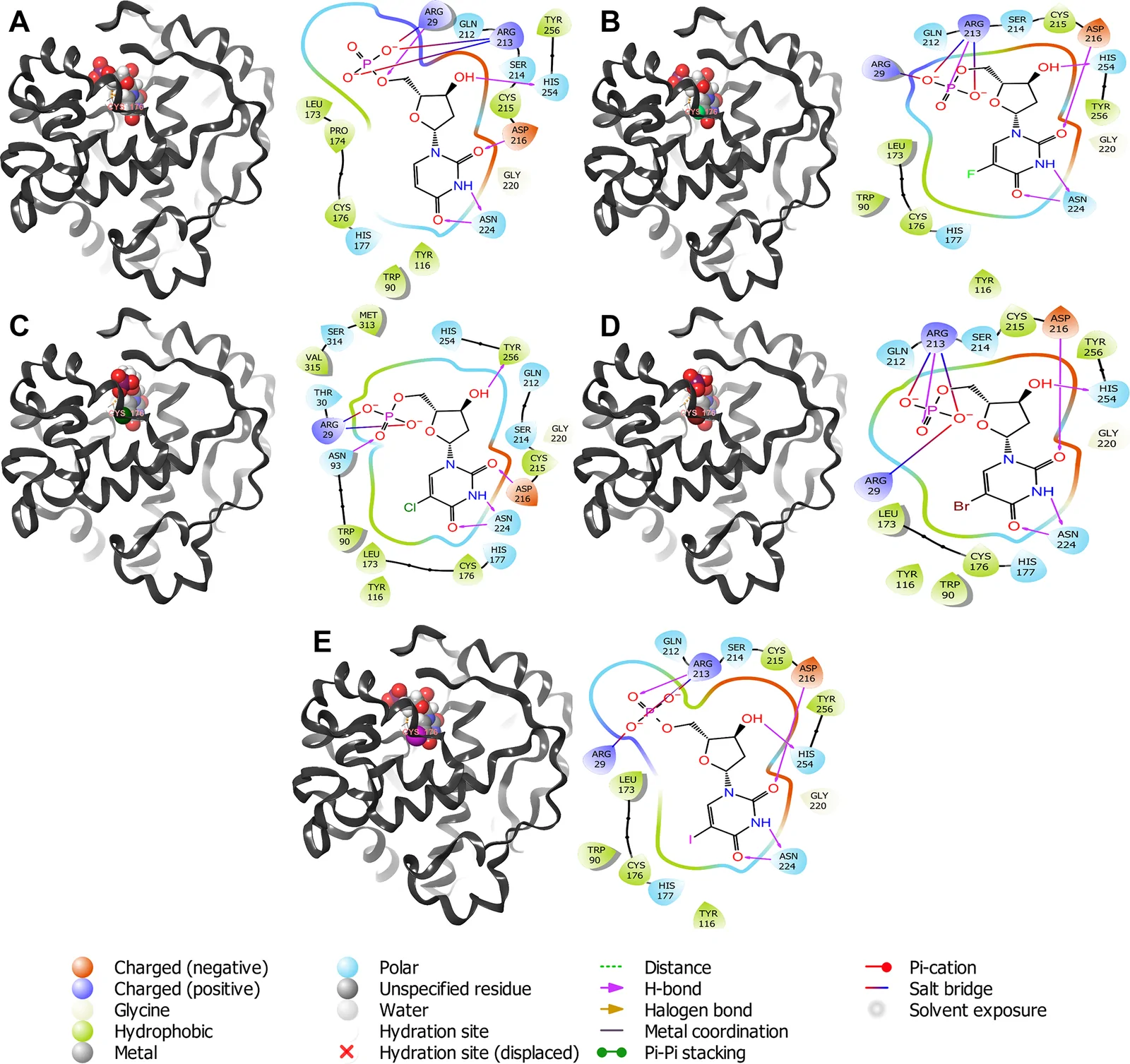
Molecular docking 3D and 2D protein-ligand interactions of dUMP (**A**), 5-fluoro-dUMP (**B**), 5-chloro-dUMP (**C**), 5-bromo-dUMP (**D**), and 5-iodo-dUMP (**E**) with thymidylate synthase of *Candida auris*.

Initially, dUMP (native ligand) and the 5-halogenated derivatives of dUMP—5-fluoro-dUMP, 5-chloro-dUMP, 5-bromo-dUMP, and 5-iodo-dUMP—were docked against the thymidylate synthase of *C. auris*. The docking scores of the complexes are present in Table S4. The docking scores of dUMP and 5-fluoro-dUMP were similar, whereas the docking scores were higher for chloro-, bromo-, and iodo-substituted derivatives, signifying a competitive inhibitory role of 5-fluoro-dUMP. For thymidylate synthase to convert dUMP to dTMP, it requires methylation on the fifth carbon of the uridine ring, which is facilitated by a sulfur atom on the Cys176 residue. In the protein-ligand interactions, dUMP shows stable binding in close proximity to the Cys176 residue, where the uridine ring’s fifth carbon is poised for a nucleophilic attack (Fig. 5). This was also observed in 5-fluoro-dUMP. However, interactions of the bulkier halogen substitutions were not in line with the Cys176 residue despite binding with strong hydrogen bonds (Fig. 5). Similar patterns of binding were also observed in docked complexes of nucleoside metabolites and thymidylate synthases of other *Candida* species like *C. albicans*, *C. glabrata*, and *C. parapsilosis* (Fig. S1 through S3). Interacting residues of dUMP and its halogenated analogs with thymidylate synthase of four *Candida* strains are present in (Table S5). The cysteine residue is conserved across the four *Candida* strains, leading to similar binding patterns (Fig. S4). Since the proteins are not experimentally solved, the amino acid sequences were used to model the proteins using AlphaFold. All four modeled thymidylate synthases were structurally valid, with no residues in disallowed regions, pLDDT confidence scores of >90, and high ERRAT quality factor (>80; Fig. S5 through S12). CastP server confirmed the presence of the binding pockets with the conserved cysteine residue in all the modeled proteins (Fig. S5 through S12).

**Fig 6:**
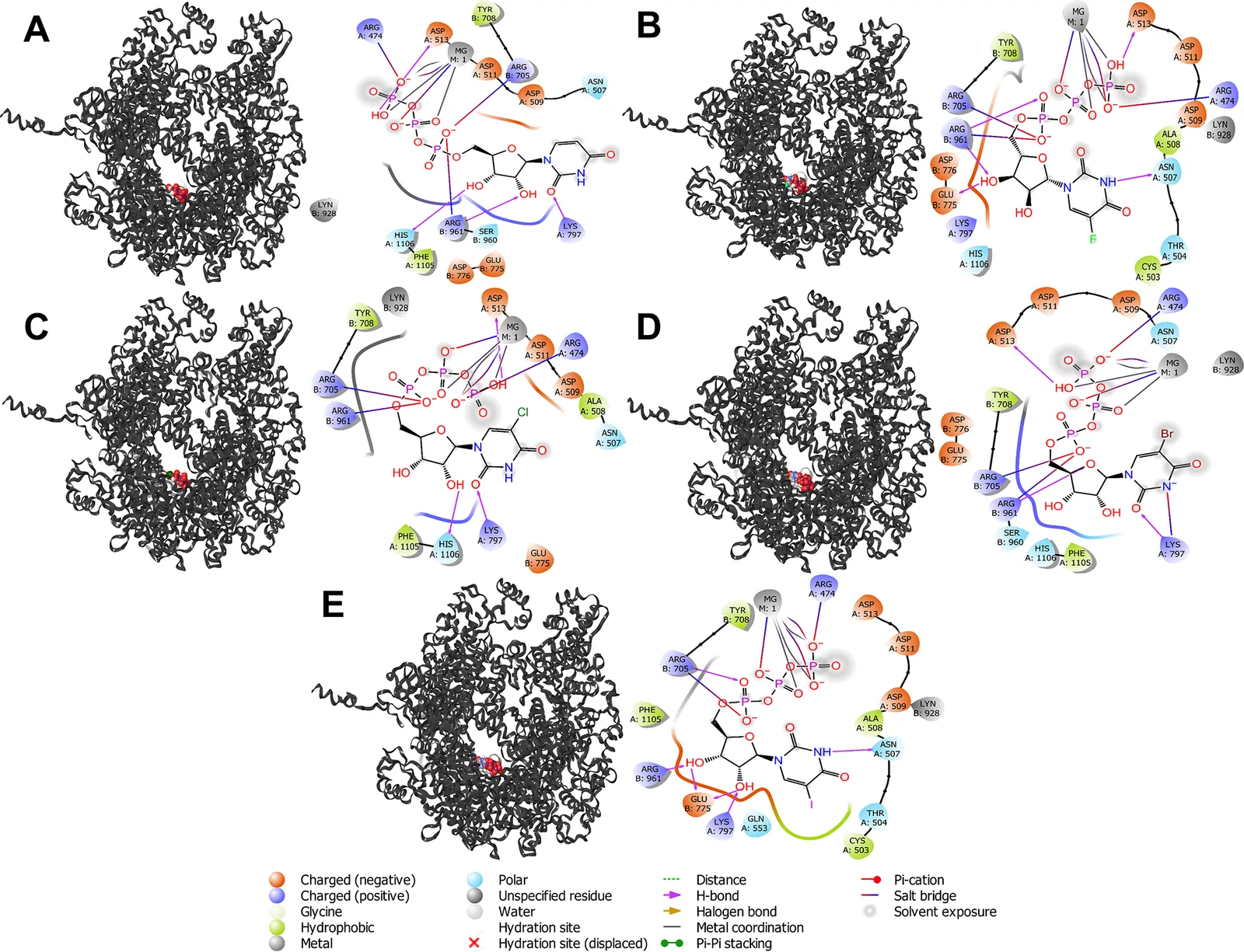
3D and 2D protein-ligand interactions of UTP (**A**), 5-fluoro-UTP (**B**), 5-chloro-UTP (**C**), 5-bromo-UTP (**D**), and 5-iodo-UTP (**E**) with RNA polymerase III of *Candida auris*.

**Fig 7:**
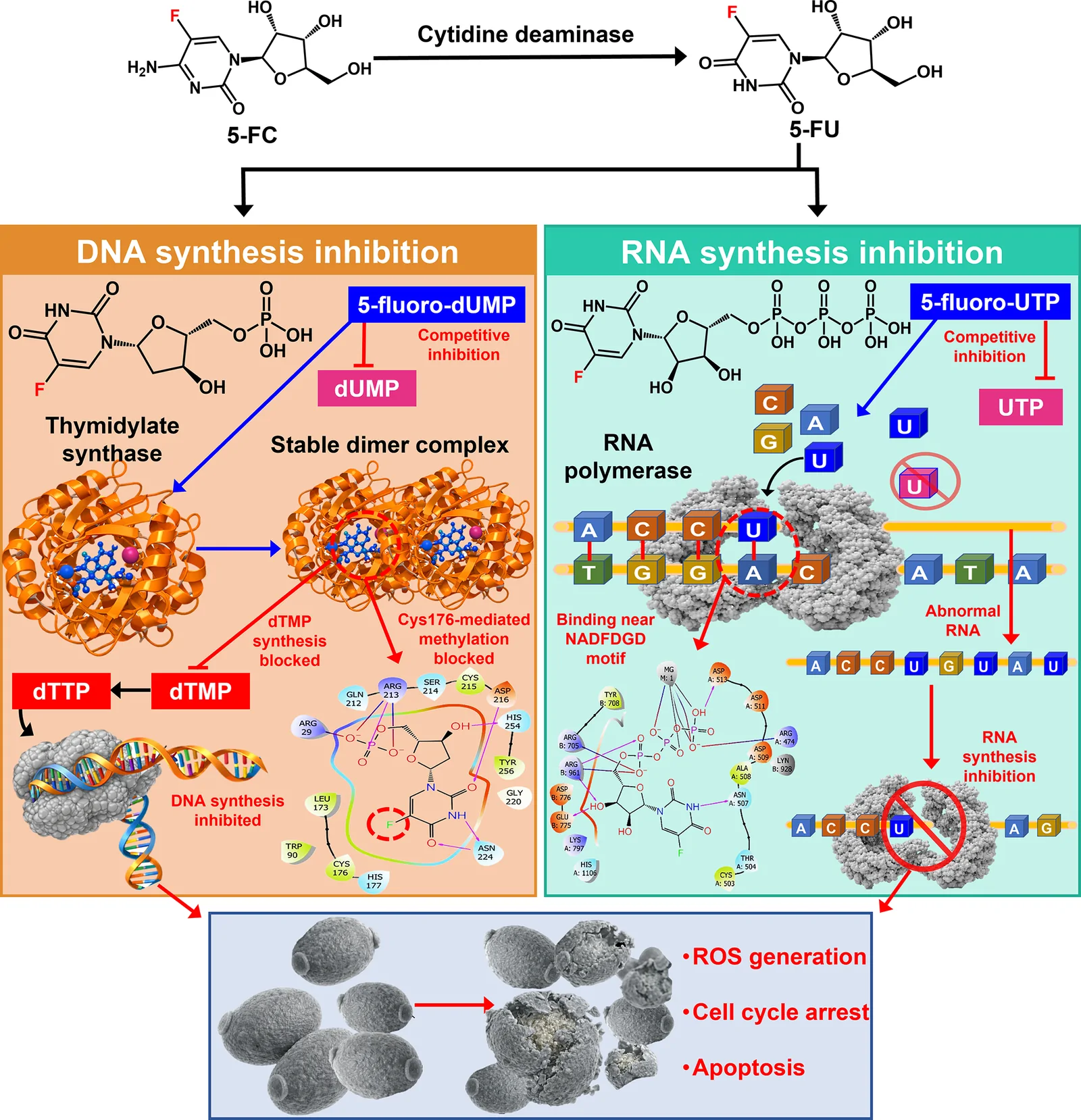
Graphical representation of the plausible mechanism of action of 5-FC and 5-FU against *Candida* species. 5-FU forms metabolites of 5-fluoro-dUMP and 5-fluoro-UTP. 5-Fluoro-dUMP inhibits thymidylate synthase by forming a stable inhibitory complex, blocking dTMP and dTTP synthesis, and suppressing DNA replication. In parallel, 5-fluoro-UTP competes with UTP and is misincorporated by RNA polymerase, generating abnormal RNA and inhibiting RNA elongation. These combined DNA and RNA synthesis defects induce replication and transcriptional stress. Downstream consequences include ROS generation, cell-cycle arrest, and apoptosis, leading to cell death.

UTP and its halogenated metabolite derivatives were docked against the RNA polymerase III elongation complex (RPC1 and RPC2) of *C. auris*. Interestingly, UTP and its halogenated counterparts had similar docking scores (Table S6). The protein-ligand interaction shows good binding interactions of all derivatives in complex pocket, with good stabilization with the Mg^2+^ metal coordination and interactions with the NADFDGD motif of the RPC1 subunit of the complex (Fig. 6). As expected, the magnesium ion forms a metal coordination bond with the phosphate group to bring the ligand closer to the NADFDGD motif (residues 507–513), wherein the aspartate trio, along with lysine, arginine, and asparagine amino acids, completes complex formation by hydrogen bonds (Fig. 6). Similar interactions were also observed when UTP and their halogenated metabolite derivatives were docked against the RNA polymerase III elongation complex (RPC1 and RPC2) of *C. albicans* (Table S6). The protein-ligand interactions also showed similar magnesium ion coordination as well as interaction with the aspartate trio of the NADFDGD motif (Fig. S13). The interacting residues of UTP and their halogenated metabolite derivatives with the RNA polymerase III elongation complex of both *C. auris* and *C. albicans* are listed in Table S7. A multiple sequence alignment was done for both RPC1 and RPC2 subunits of the four *Candida* strains, as previously done in the case of the thymidylate synthase. The percentage identity matrix shows high similarity (>80%) between the RPC1 and RPC2 subunits of the four *Candida* strains (Table S8). The NADFDGD motif was also conserved across the four strains (Fig. S14). Although the sequence similarity was high, the multimer algorithm was not able to give a reliable RPC1 and RPC2 complex for *C. glabrata* and *C. parapsilosis* (results not shown). The predicted complexes of both *C. albicans* and *C. auris* were structurally valid as evaluated using the pLDDT confidence scores and sequence coverage heatmaps (Fig. S15 and S16). The predicted complexes also had the distinctive horseshoe shape found in typical RNA polymerase elongation complexes (Fig. 6 and Fig. S13). These docking analyses partially support nucleic acid synthesis inhibition by both 5-FC and 5-FU (Fig. 7).

## DISCUSSION

The present study identifies 5-FU as a potent and mechanistically distinct antifungal agent against *C. auris* and other clinically relevant *Candida* species, addressing a critical therapeutic gap posed by multidrug resistance and limited antifungal classes. By combining phenotypic assays, metabolic rescue experiments, synergistic drug interaction studies, multi-model toxicity evaluation, and structure-guided *in silico* analysis, this work provides a comprehensive and coherent framework for understanding the antifungal potential of halogenated nucleoside mimetics.

Among the 121 nucleoside and nucleobase derivatives screened, only 5-FU and 5-FC exhibited potent antifungal activity, underscoring the exceptional specificity of C-5 fluorination on the pyrimidine ring (30). The lack of activity observed for bulkier halogen substitutions (chloro, bromo, and iodo) and ribose-modified analogs highlights the delicate balance between steric compatibility, enzymatic recognition, and intracellular activation required for antifungal efficacy (31). The hydrogen atom being replaced by the fluorine atom causes only minor changes in structure in comparison to bulkier halogens while imparting significant stereochemical properties (32, 33). This might be a potential reason for the inactivity of other halogen atom substitutions. This finding reinforces earlier observations that fluorine, due to its minimal steric footprint and strong inductive effects, uniquely preserves native substrate mimicry while introducing antimetabolite behavior. 5-FU displayed uniform low-MIC activity across all tested *Candida* species, including fluconazole-resistant *C. albicans* and *C. auris*, whereas 5-FC exhibited species-dependent variability (Table 1). Also, the cell growth and cell survival assays show higher potency of 5-FU in comparison to 5-FC (Fig. 1A, 1B and 2). This divergence likely reflects differences in cytidine deaminase activity, nucleoside transporter expression, and intracellular metabolic flux, which may be higher for 5-FU than 5-FC and vary across *Candida* species (34). These results suggest that uridine-based analogs may provide broader-spectrum antifungal coverage than cytidine analogs.

A key and clinically significant observation is the fungicidal nature of 5-fluorouridine against *C. auris*, with MIC values substantially lower than those of frontline antifungals such as caspofungin and clotrimazole (Table S2). Many current antifungals exhibit fungistatic behavior against *C. auris*, contributing to persistence and relapse. In contrast, the rapid reduction in viable CFUs upon 5-FU treatment indicates irreversible metabolic collapse, consistent with nucleic acid synthesis disruption rather than cell wall or membrane stress alone (35). The uridine rescue assay provides compelling mechanistic evidence that the antifungal activity of 5-FU is driven by competitive interference with endogenous uridine metabolism (Fig. 1C and D). Partial to near-complete growth restoration upon equimolar uridine supplementation indicates that 5-FU acts as a fraudulent nucleotide, hijacking fungal nucleic acid synthesis pathways (Fig. 7). The incomplete rescue at supra-MIC concentrations further suggests that once intracellular toxic metabolites accumulate beyond a threshold, the damage becomes irreversible, explaining the fungicidal phenotype (36, 37). Importantly, ROS measurements showed significant oxidative stress induction (Fig. 1E), a hallmark of nucleic acid synthesis inhibition (38). This supports a primary antimetabolite mechanism via nucleic acid synthesis inhibition (Fig. 7), minimizing the likelihood of off-target oxidative toxicity.

The *in silico* docking analyses provide structural context to the observed phenotypes (Fig. 5 and 6). Metabolized derivatives of 5-FU showed competitive binding within the catalytic pocket of thymidylate synthase, positioning the C-5 fluorinated uridine moiety adjacent to the conserved catalytic cysteine (Cys176; Fig. 5). This alignment prevents the essential methylation step required for dTMP synthesis, leading to thymidine starvation and DNA replication arrest (39). The inability of bulkier halogenated analogs to orient properly within this pocket rationalizes their poor antifungal activity despite reasonable docking scores (40). In parallel, docking of UTP and fluorinated UTP analogs against RNA polymerase III revealed highly conserved interactions with the NADFDGD motif and magnesium-coordinated active site, essential for transcription elongation (Fig. 6). The near-equivalent binding energies of native and fluorinated nucleotides explain why RNA polymerase readily incorporates 5-FUTP, leading to widespread RNA miscoding and transcriptional dysfunction (35). The conservation of this motif across *Candida* species further accounts for the broad antifungal spectrum of 5-FU (Fig. S14).

These findings support a dual-pathway inhibition model, where 5-FU simultaneously disrupts DNA synthesis through thymidylate synthase inhibition and RNA synthesis via RNA polymerase III misincorporation similar to 5-fluorouracil (41). Fluoropyrimidine nucleotides exert cytotoxicity through coordinated inhibition of both DNA and RNA synthesis. Formation of a stable inhibitory complex between 5-fluoro-dUMP (5-FU metabolite) and thymidylate synthase depletes dTTP pools, inducing replication stress and impairing DNA synthesis (Fig. 7). In parallel, incorporation of 5-fluoro-UTP (5-FU metabolite) into nascent RNA disrupts RNA polymerase-mediated elongation, generating aberrant, nonfunctional transcripts (Fig. 7). These DNA- and RNA-directed effects compromise genome integrity and protein biosynthesis. The combined metabolic stress can trigger oxidative stress, cell-cycle arrest, and apoptosis, ultimately culminating in cell death (Fig. 7). Such poly-target interference within essential metabolic pathways significantly raises the barrier to resistance development, particularly compared to single-target antifungals.

The demonstrated synergy between 5-FU and caspofungin, clotrimazole, and miconazole is particularly noteworthy. Echinocandins compromise cell wall integrity, while azoles disrupt ergosterol biosynthesis. When combined with nucleic acid synthesis inhibition, fungal cells face simultaneous structural and metabolic stress (Fig. 3). This multi-axis pressure likely explains the observed FICI values indicative of synergy or additivity (42). From a translational perspective, such combinations could lower required doses of existing antifungals, mitigating toxicity while overcoming intrinsic resistance (42). Given the limited antifungal pipeline for *C. auris*, repurposing nucleoside analogs as adjuvants represents a strategically attractive and clinically feasible approach.

Despite structural similarity to anticancer fluoropyrimidines, 5-FU exhibited remarkably low toxicity across plant, nematode, and human cell models, even at concentrations several orders of magnitude above antifungal MICs (Fig. 4). The absence of acute cytotoxicity in HepG2 cells and minimal effects in *C. elegans* strongly suggest preferential fungal uptake and metabolism, likely due to differences in nucleoside transporter specificity and enzymatic activation pathways, which was also observed with 5-fluorocytosine. Humans lack enzymes like cytosine deaminase and cytosine permease, which might partially explain the non-cytotoxic results of 5-FC (43). While *in silico* ADMET predictions flagged potential systemic toxicity endpoints (Table S3), experimental validation is required to conclusively prove these predictive flags. Nevertheless, these signals warrant careful dose optimization and pharmacokinetic studies in future *in vivo* models. Advanced drug-delivery strategies like nanoparticles and liposomes also aided in the targeted delivery of 5-FU and mitigating toxicity (44–46). This study primarily evaluates planktonic growth inhibition. Given the clinical importance of *C. auris* biofilms, future work should assess antibiofilm efficacy in *in vitro* models. Additionally, *in vivo* infection studies will be essential to confirm therapeutic windows, tissue distribution, and resistance emergence dynamics. Structure-guided optimization of fluorinated nucleosides, potentially through prodrug strategies or targeted delivery systems, may further enhance efficacy while preserving safety (47).

Future applications could see 5-FU integrated into bioactive coatings for medical devices to prevent fungal colonization, as it targets core metabolic pathways rather than just cell wall stress (48). Furthermore, 5-FU could be utilized to develop bio-switchable polymers for healthcare surfaces that release the nucleoside mimetic only upon detecting fungal-specific metabolic activity or pH changes (48). Its potential as a metabolic Trojan horse is supported by its ability to hijack specific fungal transporters and competitively interfere with endogenous uridine metabolism, potentially leading to applications across other fungal genera (6, 49). From a drug-design perspective, the C-5-fluorinated pyrimidine ring serves as a privileged scaffold for developing entire libraries of next-generation antimetabolites with optimized safety profiles. Furthermore, the drug’s ability to concurrently inhibit both DNA and RNA synthesis offers a strategic advantage in preventing the rapid evolutionary development of resistance (50). To advance these findings toward clinical use, future research should utilize RNA-sequencing to further map the molecular blueprint of fungal cell death (51). Beyond immediate cell death, 5-FU’s disruption of nucleic acid synthesis likely compromises the fungus’s ability to mount a rapid transcriptional response to environmental stressors or host immunity, effectively suppressing key virulence factors (50). Future studies could investigate whether sub-inhibitory doses of 5-FU can make the pathogen more susceptible to natural host clearance. These multidisciplinary applications position 5-FU not only as a standalone treatment but also as a foundation for innovative antimicrobial strategies against emerging global pathogens, especially fungi.

### Conclusion

This study identifies 5-fluorouridine as a potent and mechanistically distinct antifungal agent against *C. auris* and other clinically relevant *Candida* species. 5-FU exhibited fungicidal activity at low concentrations and demonstrated strong synergy with existing azole and echinocandin antifungals. Mechanistic analyses revealed dual disruption of DNA and RNA synthesis through inhibition of thymidylate synthase and misincorporation by RNA polymerase III. Importantly, 5-FU showed minimal toxicity across plant, nematode, and human cell lines, even at supra-MIC doses. These results support C-5-fluorinated nucleosides as promising scaffolds for repurposing and combination-based strategies to combat multidrug-resistant *Candida* infections.

## Data Availability

All data generated or analyzed during this study are included in this published article and its Supplemental material.

## SUPPLEMENTAL MATERIAL

Supplemental material aac.00027-26-s0001.docx Tables S1–S8; Fig. S1–S16.

ASM does not own the copyrights to Supplemental Material that may be linked to, or accessed through, an article. The authors have granted ASM a non-exclusive, world-wide license to publish the Supplemental Material files. Please contact the corresponding author directly for reuse.
