# Supplementary material for: Antifungal activity of the nucleoside mimetic 5-fluorouridine against Candidozyma auris and Candida species
Source: Antimicrob Agents Chemother. 2026 Aug 14;70(9):e00027-26. doi: 10.1128/aac.00027-26 (PMC13712885; doi:10.1128/aac.00027-26)
Supplement: Supplementary file 1 [file aac.00027-26-s0001.docx]

**Supplementary material**

**Antifungal activity of the nucleoside mimetic 5-fluorouridine against *Candidozyma auris* (*Candida auris*) and *Candida* species**

**Supplementary Table S1:** List of nucleoside derivatives used in the study.

|  | **Compound Name** | **CAS no.** | **Structure** |
| --- | --- | --- | --- |
| 1. | 2'-Amino-2'-deoxyuridine | 26889-39-4 |  |
| 2. | 5-Bromouridine | 957-75-5 |  |
| 3. | 2'-C-Methyluridine | 31448-54-1 |  |
| 4. | (2'R)-2'-Deoxy-2'-fluoro-2'-methyl-uridine | 863329-66-2 |  |
| 5. | 2'-Deoxy-2'-fluorouridine | 784-71-4 |  |
| 6. | 2'-Deoxy-5'-o-dmt-2'-fluorouridine | 146954-74-7 |  |
| 7. | 2'-Deoxyuridine | 951-78-0 |  |
| 8. | 5-Ethynyluridine | 69075-42-9 |  |
| 9. | 5-Fluorouridine | 316-46-1 |  |
| 10. | 5-Methyluridine | 1463-10-1 |  |
| 11. | 2',3'-O-Isopropylideneuridine | 362-43-6 |  |
| 12. | 5'-O-T-Butyldimethylsilyl 2'-deoxy-5-iodo-uridine | 134218-81-8 |  |
| 13. | 2-Thiouridine | 20235-78-3 |  |
| 14. | 2',3',5'-Triacetyluridine | 4105-38-8 |  |
| 15. | Uridine | 58-96-8 |  |
| 16. | Adenosine | 58-61-7 |  |
| 17. | 8-Bromoadenosine | 2946-39-6 |  |
| 18. | 2-Chloroadenosine | 146-77-0 |  |
| 19. | 2-Chloroadenosine hemihydrate | 81012-94-4 |  |
| 20. | 5'-Chloro-5'-deoxyadenosine | 892-48-8 |  |
| 21. | 2'-Deoxyadenosine | 958-09-8 |  |
| 22. | 2'-Deoxyadenosine monohydrate | 16373-93-6 |  |
| 23. | 2',3'-Dideoxyadenosine | 4097-22-7 |  |
| 24. | Dmt-2'fluoro-da(bz) amidite | 136834-22-5 |  |
| 25. | 2-Fluoroadenosine | 146-78-1 |  |
| 26. | 2-Iodoadenosine | 35109-88-7 |  |
| 27. | N6-Methyladenosine | 1867-73-8 |  |
| 28. | 2',3'-O-Isopropylideneadenosine | 362-75-4 |  |
| 29. | 3'-O-Methyladenosine | 10300-22-8 |  |
| 30. | 5'-S-Methyl-5'-thioadenosine | 2457-80-9 |  |
| 31. | 2',3',5'-Tri-o-acetyladenosine | 7387-57-7 |  |
| 32. | 2-Thioadenosine | 43157-50-2 |  |
| 33. | 5-Azacytidine | 320-67-2 |  |
| 34. | 5-Bromocytidine | 3066-86-2 |  |
| 35. | 5-Bromo-2'-deoxycytidine | 1022-79-3 |  |
| 36. | Cytidine | 65-46-3 |  |
| 37. | 2'-Deoxy-2'-fluorocytidine | 10212-20-1 |  |
| 38. | 2'-Deoxy-2',2'-difluorocytidine | 95058-81-4 |  |
| 39. | 2'-Deoxy-2'-fluorocytidine hydrate | 10212-20-1 |  |
| 40. | 2'-Deoxy-5-fluorocytidine | 10356-76-0 |  |
| 41. | 5'-Deoxy-5-fluorocytidine | 66335-38-4 |  |
| 42. | 5'-Deoxy-5-fluoro-n-[(pentyloxy)carbonyl]cytidine 2',3'-diacetate | 154361-50-9 |  |
| 43. | 2',3'-Di-o-acetyl-5'-deoxy-5-fluorocytidine | 161599-46-8 |  |
| 44. | 5-Fluorocytidine | 2341-22-2 |  |
| 45. | 5-Iodocytidine | 1147-23-5 |  |
| 46. | 5-Iodo-2'-deoxycytidine | 611-53-0 |  |
| 47. | 5-Methylcytidine | 2140-61-6 |  |
| 48. | 5-Methyl-2'-deoxycytidine | 838-07-3 |  |
| 49. | N4-Acetylcytidine | 3768-18-1 |  |
| 50. | N4-Acetyl-2'-deoxycytidine | 32909-05-0 |  |
| 51. | N4-Benzoyl-2'-fluoro-2'-deoxycytidine | 146954-77-0 |  |
| 52. | 2'-O-Methylcytidine | 2140-72-9 |  |
| 53. | 2'-O-(2-Methoxyethyl)-cytidine | 223777-16-0 |  |
| 54. | 2',3'-O-Isopropylidenecytidine | 362-42-5 |  |
| 55. | (2'R)-N-Benzoyl-2'-deoxy-2'-fluoro-2'-methylcytidine 3',5'-dibenzoate | 874638-98-9 |  |
| 56. | 8-Bromoguanosine | 4016-63-1 |  |
| 57. | 8-Bromoguanosine hydrate | 332359-99-6 |  |
| 58. | 6-Chloroguanosine | 2004-07-1 |  |
| 59. | Guanosine | 118-00-3 |  |
| 60. | N2-(2-Methylpropanoyl)-guanosine | 64350-24-9 |  |
| 61. | N2-Isobutyryl-2'-deoxyguanosine | 68892-42-2 |  |
| 62. | 2'-O-Methylguanosine | 2140-71-8 |  |
| 63. | 3'-O-Methylguanosine | 10300-27-3 |  |
| 64. | 2',3'-O-Isopropylideneguanosine | 362-76-5 |  |
| 65. | 8-Oxo-2'-deoxyguanosine | 88847-89-6 |  |
| 66. | 2',3',5'-Triacetylguanosine | 6979-94-8 |  |
| 67. | 5-Bromo-2'-deoxy-2'-fluoro-3',5'-di-o-acetyl uridine | 1188522-91-9 |  |
| 68. | Brivudine | 69304-47-8 |  |
| 69. | 5-Bromo-2'-deoxyuridine | 59-14-3 |  |
| 70. | 3',5'-Di-o-acetyl-5-bromo-2'-deoxy-d-uridine | 6161-23-5 |  |
| 71. | 5-Chloro-2'-deoxyuridine | 50-90-8 |  |
| 72. | 2',2'-Difluoro-2'-deoxyuridine | 114248-23-6 |  |
| 73. | 5-Iodo-2'-deoxyuridine | 54-42-2 |  |
| 74. | Fialuridine | 69123-98-4 |  |
| 75. | 2'-Deoxy-5-fluorouridine | 50-91-9 |  |
| 76. | 2'-Deoxy-5'-o-dmt-5-iodouridine | 104375-88-4 |  |
| 77. | 5-Iodo-2'-o-methyluridine | 34218-84-3 |  |
| 78. | 2-Chlorodeoxyadenosine | 4291-63-8 |  |
| 79. | 2'-Fluoro-2'-deoxyadenosine | 64183-27-3 |  |
| 80. | N6-Benzoyl-2'-fluoro-2'-deoxyadenosine | 136834-20-3 |  |
| 81. | N4-Anisoyl-2'-deoxycytidine | 48212-99-3 |  |
| 82. | 5-Ethynyl-2'-deoxycytidine | 69075-47-4 |  |
| 83. | N4-Benzoyl-5'-o-(4,4'-dimethoxytrityl)-2'-fluoro-2'-deoxycytidine | 146954-77-0 |  |
| 84. | 5'-O-Dmt-n4-benzoyl-2',2'-difluoro-2'-deoxycytidine 3'-ce phosphoramidite | 102212-98-6 |  |
| 85. | 5-Methylcyclocytidine hydrochlorine | 51391-96-9 |  |
| 86. | N-Acetyl-5'-O-(4,4-dimethoxytrityl)-2'-O-methylcytidine-3'-(2-cyanoethyl-N,N-diisopropyl)phosphoramidite | 199593-09-4 |  |
| 87. | N4-Acetyl-2'-deoxy-2'-fluorocytidine | 159414-97-8 |  |
| 88. | N-Isobutyryl-2',3'-acetyl-guanosine | 163586-86-5 |  |
| 89. | N2-Phenoxyacetylguanosine | 119824-66-7 |  |
| 90. | 8-Bromo-2'-deoxyguanosine | 13389-03-2 |  |
| 91. | 8-Hydroxy-2'-deoxyguanosine | 88847-89-6 |  |
| 92. | 5-Acetyluracil | 6214-65-9 |  |
| 93. | 6-Aminouracil | 873-83-6 |  |
| 94. | 5-Bromouracil | 51-20-7 |  |
| 95. | 5-Chlorouracil | 1820-81-1 |  |
| 96. | 6-Chlorouracil | 4270-27-3 |  |
| 97. | 5-Cyanouracil | 4425-56-3 |  |
| 98. | 5-Ethyluracil | 4212-49-1 |  |
| 99. | 5-Fluorouracil | 51-21-8 |  |
| 100. | 2-Methoxy-5-fluorouracil | 1480-96-2 |  |
| 101. | 2-Ethoxy-5-fluorouracil | 56177-80-1 |  |
| 102. | 5-Formyluracil | 1195-08-0 |  |
| 103. | 6-Iodouracil | 4269-94-7 |  |
| 104. | 5-Methoxyuracil | 6623-81-0 |  |
| 105. | 5-Bromo-6-methyluracil | 15018-56-1 |  |
| 106. | 5-(Chloromethyl)uracil | 3590-48-5 |  |
| 107. | 5-Chloro-6-methyluracil | 16018-87-4 |  |
| 108. | 6-(Chloromethyl)uracil | 18592-13-7 |  |
| 109. | 6-Chloro-3-methyluracil | 4318-56-3 |  |
| 110. | 5-(Trifluoromethyl)uracil | 54-20-6 |  |
| 111. | 6-(Trifluoromethyl)uracil | 672-45-7 |  |
| 112. | 6-Chloro-1,3-dimethyluracil | 6972-27-6 |  |
| 113. | 6-[(3-Chloropropyl)amino]-1,3-dimethyluracil | 34654-81-4 |  |
| 114. | 5-Fluoro-1,3-dimethyluracil | 3013-92-1 |  |
| 115. | 5-Hydroxymethyluracil | 4433-40-3 |  |
| 116. | Uracil | 66-22-8 |  |
| 117. | Cytosine | 71-30-7 |  |
| 118. | 5-Bromocytosine | 2240-25-7 |  |
| 119. | 5-Chlorocytosine | 2347-43-5 |  |
| 120. | 5-Fluorocytosine | 2022-85-7 |  |
| 121. | 5-Iodocytosine | 1122-44-7 |  |

**Supplementary Table S2:** MICs of clinical antifungals against *C. auris*.

| **Compounds** | ***C. auris* 17809 (µg/mL)** | **Structure** | **Antifungal** |
| --- | --- | --- | --- |
| Amphotericin B | 400 |  | Polyenes |
| Clotrimazole | 10 |  | Azole Compounds |
| Posaconazole | >200 |  |  |
| Isavuconazole | 500 |  |  |
| Econazole nitrate | 6 |  |  |
| Miconazole nitrate | 3 |  |  |
| Fluconazole | 100 |  |  |
| Butoconazole | 4 |  |  |
| Anidulafungin | 50 |  | Echinocandins |
| Caspofungin | 10 |  |  |
| Micafungin | 50 |  |  |
| Terbinafine hydrochloride | >500 |  |  |

**Supplementary Table S3.** *In silico* adsorption, distribution, metabolism and toxicity (ADMET) analysis of 5-fluorocytidine (5-FC) and 5-fluorourdine (5-FU).

| **Endpoint** | **5-FC** | **5-FU** |
| --- | --- | --- |
| Toxicity | | |
| Hepatotoxicity | Active | Active |
| Neurotoxicity | Active | Active |
| Nephrotoxicity | Active | Active |
| Respiratory toxicity | Active | Active |
| Cardiotoxicity | Inactive | Inactive |
| Carcinogenicity | Active | Inactive |
| Immunotoxicity | Inactive | Inactive |
| Mutagenicity | Inactive | Inactive |
| Cytotoxicity | Inactive | Inactive |
| Ecotoxicity | Inactive | Inactive |
| Tox-21 parameters | | |
| Aryl hydrocarbon Receptor (AhR) | Inactive | Inactive |
| Androgen Receptor (AR) | Inactive | Inactive |
| Androgen Receptor Ligand Binding Domain (AR-LBD) | Inactive | Inactive |
| Aromatase | Inactive | Inactive |
| Estrogen Receptor Alpha (ER) | Inactive | Inactive |
| Estrogen Receptor Ligand Binding Domain (ER-LBD) | Inactive | Inactive |
| Peroxisome Proliferator Activated Receptor Gamma (PPAR-Gamma) | Inactive | Inactive |
| Nuclear factor (erythroid-derived 2)-like 2/antioxidant responsive element (nrf2/ARE) | Inactive | Inactive |
| Heat shock factor response element (HSE) | Inactive | Inactive |
| Mitochondrial Membrane Potential (MMP) | Inactive | Inactive |
| ATPase family AAA domain-containing protein 5 (ATAD5) | Inactive | Inactive |
| Thyroid hormone receptor alpha (THRα) | Inactive | Inactive |
| Thyroid hormone receptor beta (THRβ) | Inactive | Inactive |
| Transtyretrin (TTR) | Inactive | Inactive |
| Ryanodine receptor (RYR) | Inactive | Inactive |
| GABA receptor (GABAR) | Inactive | Inactive |
| Glutamate N-methyl-D-aspartate receptor (NMDAR) | Inactive | Inactive |
| alpha-amino-3-hydroxy-5-methyl-4-isoxazolepropionate receptor (AMPAR) | Inactive | Inactive |
| Kainate receptor (KAR) | Inactive | Inactive |
| Achetylcholinesterase (AChE) | Inactive | Inactive |
| Constitutive androstane receptor (CAR) | Inactive | Inactive |
| Pregnane X receptor (PXR) | Inactive | Inactive |
| NADH-quinone oxidoreductase (NADHOX) | Inactive | Inactive |
| Voltage gated sodium channel (VGSC) | Inactive | Inactive |
| Na+/I- symporter (NIS) | Inactive | Inactive |
| Cytochrome CYP1A2 | Inactive | Inactive |
| Cytochrome CYP2C19 | Inactive | Inactive |
| Cytochrome CYP2C9 | Inactive | Inactive |
| Cytochrome CYP2D6 | Inactive | Inactive |
| Cytochrome CYP3A4 | Inactive | Inactive |
| ADME | | |
| BBB | 0.31367 | 0.49036 |
| Buffer_solubility_mg_L | 34778.3 | 14806.5 |
| Caco2 | 20.2558 | 19.8859 |
| HIA | 32.1051 | 30.4478 |
| Pgp_inhibition | Non | Non |
| Plasma_Protein_Binding | 4.45591 | 8.90193 |
| Pure_water_solubility_mg_L | 57820.1 | 141255 |
| Skin_Permeability | -5.26349 | -5.2152 |
| SKlogP_value | -2.9978 | -2.44418 |
| Rule_of_Five | Suitable | Suitable |
| Rule_of_Five_Violations | 0 | 0 |
| WDI_like_Rule | In 90% cutoff | In 90% cutoff |
| WDI_like_Rule_Violations | 0 | 0 |

**Supplementary Table S4.** Docking scores (kcal/mol) of dUMP and its halogenated analogs with thymidylate synthase of *C. albicans*. The docking scores were consistent across multiple independent runs.

| **Compound** | ***C. albicans*** | ***C. glabrata*** | ***C. parapsilosis*** | ***C. auris*** |
| --- | --- | --- | --- | --- |
| dUMP | -9.262 | -6.863 | -8.64 | -8.408 |
| 5-Fluoro-dUMP | -9.242 | -4.852 | -6.501 | -8.356 |
| 5-Chloro-dUMP | -8.345 | -3.343 | -5.346 | -7.812 |
| 5-Bromo-dUMP | -7.432 | -3.441 | -4.645 | -7.555 |
| 5-Iodo-dUMP | -7.176 | -1.839 | -3.551 | -7.05 |

**Supplementary Table S5.** Interacting residues of dUMP and its halogenated analogs with thymidylate synthase of 4 *Candida* strains.

| **Strain** | **dUMP** | **5-fluoro-dUMP** | **5-Chloro-dUMP** | **5-Bromo-dUMP** | **5-Iodo-dUMP** |
| --- | --- | --- | --- | --- | --- |
| ***C. albicans*** | ARG29, ARG213,  SER214, ASP216, ASN224, TYR256 | ARG29,  ARG213,  SER214,  ASP216,  ASN224,  HIS254 | ARG29,  ILE89,  ASN93,  ASP216,  ASN224 | ARG29,  ARG213,  ASP216,  ASN224,  TYR256 | ARG29,  ASN93,  ASP216, ASN224,  TYR256 |
| ***C. glabrata*** | ARG28, ARG200, SER201, ASP203, ASN211, HIS241 | ARG28,  TYR115,  HIS176,  ARG200,  SER201,  ASN211 | ARG28,  TYR 115,  ARG200,  SER201,  ASP203,  ASN211,  HIS241 | ARG28,  ASN92,  ASN211,  TYR243 | ARG28, ARG200, SER201, ASN211, HIS241 |
| ***C. parapsilosis*** | ARG29, ARG219, ASP222, ASN230, HIS260, TYR262 | ARG29,  ASN230,  SER320, | ARG29,  SER220,  ASP222,  ASN230,  SER320 | ARG29,  TYR116,  ASN230,  TYR262,  SER320 | TYR116, SER220, ASP222, ASN230, SER320 |
| ***C. auris*** | ARG29, ARG213, ASP216, ASN224, HIS254 | ARG29,  ARG213,  ASP216,  ASN224,  HIS254 | ARG29,  ASN93,  ASP216,  ASN224,  TYR256 | ARG29,  ARG213,  ASP216,  ASN224,  HIS254 | ARG29, ARG213, ASP216, ASN224, HIS254 |

**Supplementary Table S6.** Docking scores (kcal/mol) of UTP and its halogenated analogs with RNA polymerase III (complex of subunits RPC1 and RPC2subunits RPC1 and RPC2) of *C. albicans* and *C. auris.* The docking scores were consistent across multiple independent runs.

| **Compound** | ***C. albicans*** | ***C. auris*** |
| --- | --- | --- |
| UTP | -12.581 | -12.425 |
| 5-Fluoro-UTP | -12.291 | -12.29 |
| 5-Chloro-UTP | -12.228 | -12.408 |
| 5-Bromo-UTP | -12.098 | -12.329 |
| 5-Iodo-UTP | -12.028 | -12.145 |

**Supplementary Table S7.** Interacting residues of UTP and its halogenated analogs with RNA polymerase III (complex of subunits RPC1 and RPC2subunits RPC1 and RPC2) of 2 *Candida* strains.

| **Strain** | **UTP** | **5-fluoro-UTP** | **5-Chloro- UTP** | **5-Bromo- UTP** | **5-Iodo- UTP** |
| --- | --- | --- | --- | --- | --- |
| ***C. albicans*** | ASP507  TYR709  GLN716  LYS921  LYS929  LYS1044 | ASP511  GLU512  GLY927  LYS921  LYS929  LYS1044 | ARG374  ARG472  GLN473  ASP509  GLU512  TYR709  LYS921  LYS929  LYS1044 | ARG706  TYR709  GLN716  LYS921  LYS929  LYS1044 | ASP509  ASP511  GLU512  TYR709  LYS921  LYS929  LYS1044 |
| ***C. auris*** | ARG474  ASP513  ARG705  LYS797  ARG961  HIS1106 | ARG474  ASN507  ASP513  ARG705  GLU775  ARG961 | ARG474  ASP513  ARG705  LYS797  ARG961  HIS1106 | ARG474  ASP513  ARG705  LYS797  ARG961 | ARG474  ASN507  ARG705  GLU775  LYS797  ARG961 |

**Supplementary Table S8.** Percent identity matrices of protein sequences of RNA polymerase III-RPC1 and RPC2 subunits of the 4 *Candida* strains.

| **RNA polymerase III RPC1 subunit** | | | | |
| --- | --- | --- | --- | --- |
| *C. glabrata* | 100 | 75.5 | 76.11 | 76.24 |
| *C. auris* | 75.5 | 100 | 82.73 | 82.32 |
| *C. albicans* | 76.11 | 82.73 | 100 | 87.08 |
| *C. parapsilosis* | 76.24 | 82.32 | 87.08 | 100 |
| **RNA polymerase III RPC2 subunit** | | | | |
| *C. glabrata* | 100 | 80.81 | 83.11 | 83.1 |
| *C. auris* | 80.81 | 100 | 85.7 | 86.42 |
| *C. albicans* | 83.11 | 85.7 | 100 | 91.91 |
| *C. parapsilosis* | 83.1 | 86.42 | 91.91 | 100 |

**Supplementary Figure S1.** 3D and 2D protein-ligand interactions of dUMP (A), 5-Fluoro-dUMP (B), 5-Chloro-dUMP (C), 5-Bromo-dUMP (D) and 5-Iodo-dUMP (E) with thymidylate synthase of *C. albicans*.

**Supplementary Figure S2.** 3D and 2D protein-ligand interactions of dUMP (A), 5-Fluoro-dUMP (B), 5-Chloro-dUMP (C), 5-Bromo-dUMP (D) and 5-Iodo-dUMP (E) with thymidylate synthase of *C. glabrata*.

**Supplementary Figure S3.** 3D and 2D protein-ligand interactions of dUMP (A), 5-Fluoro-dUMP (B), 5-Chloro-dUMP (C), 5-Bromo-dUMP (D) and 5-Iodo-dUMP (E) with thymidylate synthase of *C. parapsilosis*.

**Supplementary Figure S4.** Multiple sequence alignment of thymidylate synthase protein sequences of KAL1578858.1 – *C. albicans*, QEO22922.1-*C. auris*, KTB11516.1- *C. glabrata* and KAI5910523.1- *C. parapsilosis.* Active site residues signified by red box, are conserved across secies and all the sequences have the important cysteine residue.

**Supplementary Figure S5.** Alphafold server entry and structural validation of the thymidylate synthase of *C. auris* (Alphafold A0A0L0P707) (A). CastP results of thymidylate synthase of *C. auris* (B) show out of 10 pockets **-** pocket 1 has favorable binding pocket- (Volume 309 A^3^. Active site residue Cysteine 176 is present as interacting residue in the binding pocket.

**Supplementary Figure S6.** Protein quality assessment of the modelled thymidylate synthase of *C. auris* using ramachandran plot (A), PROCHECK summary (B) and ERRAT analysis (C).

**Supplementary Figure S7.** Alphafold server prediction and structural validation of the thymidylate synthase of *C. parapsilosis* (A). CastP results of thymidylate synthase of *C. parpasilosis* (B) show out of 10 pockets **-** pocket 1 has favorable binding pocket- (Volume 309 A^3^. Active site residue Cysteine 176 is present as interacting residue in the binding pocket.

**Supplementary Figure S8.** Protein quality assessment of the modelled thymidylate synthase of *C. parapsilosis* using ramachandran plot (A), PROCHECK summary (B) and ERRAT analysis (C).

**Supplementary Figure S9.** Alphafold server prediction and structural validation of the thymidylate synthase of *C. glabrata* (A). CastP results of thymidylate synthase of *C. glabrata* (B) show out of 10 pockets **-** pocket 1 has favorable binding pocket- (Volume 380 A^3^. Active site residue Cysteine 175 is present as interacting residue in the binding pocket.

**Supplementary Figure S10.** Protein quality assessment of the modelled thymidylate synthase of *C. glabrata* using ramachandran plot (A), PROCHECK summary (B) and ERRAT analysis (C).

**Supplementary Figure S11.** Alphafold server prediction and structural validation of the thymidylate synthase of *C. albicans* (A). CastP results of thymidylate synthase of *C. albicans* (B) show out of 10 pockets **-** pocket 1 has favorable binding pocket- (Volume 643 A^3^. Active site residue Cysteine 176 is present as interacting residue in the binding pocket.

**Supplementary Figure S12.** Protein quality assessment of the modelled thymidylate synthase of *C. albicans* using ramachandran plot (A), PROCHECK summary (B) and ERRAT analysis (C).

**Supplementary Figure S13.** 3D and 2D protein-ligand interactions of UTP (A), 5-Fluoro-UTP (B), 5-Chloro- UTP (C), 5-Bromo- UTP (D) and 5-Iodo- UTP (E) with RNA polymerase III (complex of subunits RPC1 and RPC2) of *C. albicans*.

**Supplementary Figure S14.** Multiple sequence alignment of RNA polymerase III subunit RPC1 protein sequences of KHC86330.1-*C. albicans*, KAM3121573.1-*C. auris*, SCV13246.1- *C. glabrata* and KAI5908503.1- *C. parapsilosis.* Active site residues signified by red box, are conserved across species and signifying the DxDGD motif. RPC1 subunit has the major catalytic motif NADFDGD motif and coordinates an Mg^2+^ ion. The second Mg^2+^ ion is coordinated by the RPC2 subunit as part of the complete enzyme complex for elongation.

**Supplementary Figure S15.** Per residue pIDDT confidence scores of the modelled RNA polymerase III (complex of subunits RPC1 and RPC2) of *Candida auris*. Sequence coverage heatmap of the modelled RNA polymerase III (complex of subunits RPC1 and RPC2) of *Candida auris*.

**Supplementary Figure S16.** Per residue pIDDT confidence scores of the modelled RNA polymerase III (complex of subunits RPC1 and RPC2) of *C. albicans*. Sequence coverage heatmap of the modelled RNA polymerase III (complex of subunits RPC1 and RPC2) of *C. albicans*.
